# From Learning to Consciousness: An Example Using Expected Float Entropy Minimisation

**DOI:** 10.3390/e21010060

**Published:** 2019-01-13

**Authors:** Jonathan W. D. Mason

**Affiliations:** Mathematical Institute, University of Oxford, Oxford OX2 6GG, UK; jonathan.mason@maths.ox.ac.uk; Tel.: +44-186-528-0615

**Keywords:** float entropy, consciousness and relationships, typical data, structures implied by neural networks

## Abstract

Over recent decades several mathematical theories of consciousness have been put forward including Karl Friston’s Free Energy Principle and Giulio Tononi’s Integrated Information Theory. In this article we further investigate theory based on Expected Float Entropy (EFE) minimisation which has been around since 2012. EFE involves a version of Shannon Entropy parameterised by relationships. It turns out that, for systems with bias due to learning, certain choices for the relationship parameters are isolated since giving much lower EFE values than others and, hence, the system defines relationships. It is proposed that, in the context of all these relationships, a brain state acquires meaning in the form of the relational content of the associated experience. EFE minimisation is itself an association learning process and its effectiveness as such is tested in this article. The theory and results are consistent with the proposition of there being a close connection between association learning processes and the emergence of consciousness. Such a theory may explain how the brain defines the content of consciousness up to relationship isomorphism.

## 1. Introduction

Consciousness is awash with relationships and associations which appear to be a fundamental aspect of conscious experience given that, for example, the colour red would lose much of its meaning if we couldn’t discern its relationship to the colour blue, a glass of water, the sound of a piano or anything else. Moreover, mathematics is also awash with relationships and this suggests a mathematical theory for how the brain defines the relational content of consciousness could well be possible. This is the objective of the article Quasi-Conscious Multivariate Systems (see reference [1]) published in 2016 which greatly developed the theory first mentioned in reference [2] and is based on expected float entropy minimisation, the definition of which is given below. The theory also has potential applications in areas such as artificial intelligence and mutual or effective interaction analysis in nerve fibres; see reference [3].

In the present article we investigate the coincidence that whilst expected float entropy minimisation was developed as a way to uncover the relationships systems define, it is itself a learning process and this fact (at least in the context of the theory presented in [1]) emphasises the relevance of association learning processes to the emergence of consciousness; for example see [4]. Furthermore, for brain structures involved in generating consciousness that are configured by a biologically implemented association learning process, it is interesting to consider the extent to which the learning process and expected float entropy minimisation are analogous; [5]. In the present article we focus on testing expected float entropy minimisation to see how effective an association learning process it actually is. We already know from [1] that expected float entropy minimisation uncovers relationships that systems define but we wish to test the extent and significance of the relationships uncovered. To do this we apply expected float entropy minimisation to a set of data to obtain relationships and then apply the relationships learned to a data completion task that is analogous to the brain’s ability for filling in missing information. This is achieved by applying the theory in reverse by going from relationships to data instead of from data to relationships. The performance is then compared with a state of the art black box using the neural fitting tool in MATLAB which is applied to the same data completion task. We will now recall the basics of the theory presented in [1].

Examples of relationships present in consciousness include: the relationships between the locations in the field of view with adjacent locations being strongly related, giving geometry; colours being strongly related such as red and burgundy or unrelated such as red and blue; similar examples involving sounds or smells or tastes or locations of touch; at a higher level of meaning involving several brain regions, objects being strongly related to particular colours, textures, places, nouns and other objects. It appears that every conscious experience involves relationships without which there would be little if any meaning.

The theory in [1] provides a starting point for a mathematical theory of how the brain defines all of the relationships underlying consciousness. It proposes that a brain state in the context of all these relationships, defined by the brain, has meaning in the form of the relational content of the associated experience. If successful, such a theory would ultimately show how the brain defines the content of consciousness up to relationship isomorphism. See Section 4.3 for a discussion on relationship isomorphism.

The issue of how a system such as the brain defines relationships is crucial. Importantly, various brain regions determine an inherent probability distribution (ergodic process) over their set of states which is not uniform due to learning processes that weaken or strengthen synapses. Hebb’s principle and its modern refinements are potentially examples of such learning processes and say that neurons increase the strength of their connections to neurons they are significantly influencing; see [6,7,8]. Therefore, the brain is biased toward certain states as a result of a long history of sensory input and is not merely driven by the senses (also see [9]). Because the system itself determines an inherent probability distribution over its states, the system can define expected quantities.

Expected float entropy can be thought of as a form of Shannon entropy parameterised by relationships where the float entropy of a state of the system is a measure of the amount of information required, in addition to the information given by the relationship parameters, in order to specify that state. Therefore, the relationship parameters that minimise the expected float entropy of the system are (according to the theory) the relationships that the system defines. According to the theory all of the relationships are defined across temporal moments and this applies, for example, to both relationships that give the underlying geometry of the field of view and the relationships between colours. The relationships provide a context relative to which the current state of the system has meaning. Whilst some aspects of consciousness are present at a given moment and some are not (which depends on the current state of the system) the context of underlying relationships defined by the system is always present, at least whilst the brain’s structure and functionality is stable. If the brain or some local region of the brain contributing to consciousness changes how it is functioning then this will change its inherent probability distribution over its states which will then change the relationships that minimise expected float entropy and consequently, according to the theory, the relational content of the experience. Such changes in functionality will occur due to the brain’s plasticity but also when activity patterns change as we transition from being awake to being in a deep sleep to being in REM sleep where activity patterns return to being similar to when awake. We recall the definition of float entropy in Section 1.1 and the definition of multi-relational float entropy in Section 4.

### 1.1. Definitions and Theory

In this subsection we provide the main definitions as previously given in [1]. Systems such as the brain, and its various regions, are networks of interacting nodes. In the case of the brain we may take the nodes of the system to be the individual neurons or possibly larger structures such as cortical columns or tuples of neurons. The nodes of the system have a repertoire (range) of states that they can be in. For example, the states that neurons can be in could be associated with different firing frequencies where as the states of tuples of neurons could be given by the aggregate of the states of the neurons of the same tuple. In the present article we assume that the node repertoire is finite (as was assumed in [1]), and the state of the system is the aggregate of the states of the nodes. In Definition 1 the elements of the set *S* are to be taken as the nodes of the system. Also see the Appendix A of notation.

**Definition** **1.** *Let S be a nonempty finite set of system nodes, n:=#S. Then for S, a* data element *representation of a system state is a set (having a unique arbitrary index label i)*

$$\begin{array}{c} S_{i}:=\left\{\left(a,f_{i}\left(a\right)\right):a\in S,\;f_{i}:S\to V\right\},\;where\;f_{i}\;is\;a\;map,V:=\left\{v_{1},v_{2},\ldots ,v_{m}\right\}is\;the\mathrm{node}\;\mathrm{repertoire} \end{array}$$

*and $f_{i}\left(a\right)$ is the state node a∈S is in when the system is in state $S_{i}$. The set of all data elements for S given V is $\Omega_{S,V}$ so that $\#\Omega_{S,V}=m^{n}$. For temporally well spaced observations, it is assumed that a given finite system defines a random variable with probability distribution $P:\Omega_{S,V}\to \left[0,1\right]$ for some finite set S and node repertoire V. If T is a finite set of numbered observations of the system then T is called the* typical data *for S. The elements of T (called* typical data elements*) are handled using a function*

$$\begin{array}{c} \tau :\left\{1,\ldots ,\#T\right\}\to \left\{i:S_{i}\in \Omega_{S,V}\right\}, \end{array}$$

*where $S_{\tau (k)}$ is the data element representation of observation number k for k∈{1,…,#T}. In particular, the function τ need not be injective since small systems may be in the same state for several observations.*

**Remark** **1.** *Note that P in Definition 1 extends to a probability measure on the power set $2^{\Omega_{S,V}}$ of $\Omega_{S,V}$ by defining*

$$\begin{array}{c} P\left(A\right):=\sum_{S_{i}\in A}P\left(S_{i}\right),\;for\;A\in 2^{\Omega_{S,V}}. \end{array}$$

*Hence, we have a probability space $\left(\Omega_{S,V},2^{\Omega_{S,V}},P\right)$ with sample space $\Omega_{S,V}$, sigma-algebra $2^{\Omega_{S,V}}$, and probability measure P. For clarification, A is just a subset of $\Omega_{S,V}$.*

We now need the definition of a weighted relation.

**Definition** **2** (Weighted relations)**.** *Let S be a nonempty set. A* weighted relation *on S is a function of the form*

$$\begin{array}{c} R:S^{2}\to \left[0,1\right], \end{array}$$

*where [0,1] is the unit interval. We say that R is:*

*1.*  reflexive *if R(a,a)=1 for all a∈S;*
*2.*  symmetric *if R(a,b)=R(b,a) for all a,b∈S.*

*The set of all reflexive, symmetric weighted-relations on S is denoted $\Psi_{S}$.*

**Remark** **2.** *Except where stated, the weighted relations used in the present article are reflexive and symmetric. The formulation of the theory allows the symmetric condition to be dropped but when relationships (between nodes for example) are anticipated to be symmetric (such as in the case of relationships that give the geometry of Euclidean space), restricting to symmetric relations reduces the dimensionality which is computationally desirable when calculating efe values. Relative to a weighted relation, the value R(a,b) quantifies the strength of the relationship between a and b, interpreted in accordance with the usual order structure on [0,1] so that R(a,b)=1 is a maximum. For a small finite set, it is useful to display a weighted relation on that set as a weighted relation table (i.e., as a matrix). An example is given in Table 1.*

Before giving the definition of float entropy we require Definitions 3 and 4.

**Definition** **3.** *Let S be as in Definition 1 and let $U:V^{2}\to \left[0,1\right]$ be a reflexive, symmetric weighted-relation on the node repertoire V; i.e., $U\in \Psi_{V}$. Then, for each data element $S_{i}\in \Omega_{S,V}$, we define a function of the form $R\left\{U,S_{i}\right\}:S^{2}\to \left[0,1\right]$ by setting*

$$\begin{array}{c} R\left\{U,S_{i}\right\}\left(a,b\right):=U\left(f_{i}\left(a\right),f_{i}\left(b\right)\right)\;for\;all\;a,b\in S, \end{array}$$

*where $f_{i}$ is the mapping associated with $S_{i}$ as in Definition 1. It is easy to see that $R\left\{U,S_{i}\right\}\in \Psi_{S}$.*

**Definition** **4.** *Let S be a nonempty finite set. Every weighted relation on S can be viewed as a $\#S^{2}$-dimensional real vector. Hence, the $d_{n}$ metric is a metric on the set of all such weighted relations by setting*

$$\begin{array}{c} d_{n}\left(R,R^{\prime }\right):={\left(\sum_{\left(a,b\right)\in S^{2}}{\left|R\left(a,b\right)-R^{\prime }\left(a,b\right)\right|}^{n}\right)}^{1/n}, \end{array}$$

*where R and $R^{\prime }$ are any two weighted relations on S. Similarly we have the metric*

$$\begin{array}{c} d_{\infty }\left(R,R^{\prime }\right):=\max_{S^{2}}\left|R\left(a,b\right)-R^{\prime }\left(a,b\right)\right|. \end{array}$$

**Definition** **5** (Float entropy)**.** *Let S be as in Definition 1, let $U\in \Psi_{V}$, and let $R\in \Psi_{S}$. The* float entropy *of a data element $S_{i}\in \Omega_{S,V}$, relative to U and R, is defined as*

$$\begin{array}{c} \mathrm{fe}\left(R,U,S_{i}\right):=\log_{2}\left(\#\left\{S_{j}\in \Omega_{S,V}:d\left(R,R\left\{U,S_{j}\right\}\right)\leq d\left(R,R\left\{U,S_{i}\right\}\right)\right\}\right), \end{array}$$

*where, in the present article (unless otherwise stated), d is the $d_{1}$ metric. Furthermore, let $P:\Omega_{S,V}\to \left[0,1\right]$ and T be as in Definition 1. The* expected float entropy*, relative to U and R, is defined as*

$$\begin{array}{c} \mathrm{efe}\left(R,U,P\right):=\sum_{S_{i}\in \Omega_{S,V}}P\left(S_{i}\right)\mathrm{fe}\left(R,U,S_{i}\right). \end{array}$$

*The efe(R,U,T) approximation of efe(R,U,P) is defined as*

$$\begin{array}{c} \mathrm{efe}\left(R,U,T\right):=\frac{1}{\#T}\sum_{k=1}^{\#T}\mathrm{fe}\left(R,U,S_{\tau (k)}\right), \end{array}$$

*where τ need not be injective by Definition 1. By construction, efe is measured in bits per data element (bpe).*

It is proposed that a system (such as the brain and its subregions) will define *U* and *R* (up to a certain resolution) under the requirement that the efe is minimised. Hence, for a given system (i.e., for a fixed *P*), we attempt to find solutions in *U* and *R* to the equation

(1) $$\begin{array}{c} \mathrm{efe}\left(R,U,P\right)=\min_{R^{\prime }\in \Psi_{S},\;U^{\prime }\in \Psi_{V}}\mathrm{efe}\left(R^{\prime },U^{\prime },P\right). \end{array}$$

In practice we replace efe(·,·,P) in Equation (1) with efe(·,·,T).

Ultimately by minimising efe in *R* and *U* we are finding relationships that have particular relevance to the more probable states of the system whilst avoiding relationships with relevance to large numbers of arbitrary improbable states of the system. Note that the ‘less than or equal to’ sign in the definition of float entropy avoids trivial solutions to Equation (1) such as when *R* and *U* are the constant functions that everywhere take the value 1.

Shannon entropy is notably used in recent theories of consciousness and self-organization; see [10,11]. The definition of float entropy (see Definition 5) has some similarity to that of Boltzmann’s entropy where the entropy of a macrostate is proportional to the log of the number of microstates satisfying the macrostate. In the case of the float entropy of a system state, the macroscopic condition is the extent to which the system state adheres to the weighted relation parameters. Whilst not to be confused with Shannon entropy, expected float entropy, efe, does have some similarities with Shannon entropy and conditional Shannon entropy in particular. Indeed, efe is a measure (in bits per data element) of the expected amount of information needed, to specify the state of the system, beyond what is already known about the system from the weighted relations provided. Shannon entropy is a measure of information content in data. As data becomes more random, Shannon entropy increases because structure in data is actually a form of redundancy. By solving Equation (1) for a given system we obtain a structure in the form of weighted relations defined by the system. Relative to these weighted relations, if the system was to become more random then the efe value for the system would increase. In order to make the similarities between efe and Shannon entropy (and in particular conditional Shannon entropy) clearer, consider the summation

(2) $$\begin{array}{c} \sum_{S_{i}\in \Omega_{S,V}}P\left(S_{i}\right)\log_{2}\left(\frac{1}{P(S_{i}|A_{S_{i}})}\right), \end{array}$$

where $A_{S_{i}}:=\left\{S_{j}\in \Omega_{S,V}:d\left(R,R\left\{U,S_{j}\right\}\right)\leq d\left(R,R\left\{U,S_{i}\right\}\right)\right\}$. The summation in (2) is similar in form to the definition of conditional Shannon entropy. Furthermore, (2) can be written as

(3) $$\begin{array}{c} \sum_{S_{i}\in \Omega_{S,V}}P\left(S_{i}\right)\log_{2}\left(\frac{\sum_{S_{j}\in A_{S_{i}}}P\left(S_{j}\right)}{P(S_{i})}\right), \end{array}$$

and, when the probabilities in the argument of the logarithm are comparable, this will give a value similar to efe(R,U,P). Finally, we can write (3) as

(4) $$\begin{array}{c} H+\sum_{S_{i}\in \Omega_{S,V}}P\left(S_{i}\right)\log_{2}\left(\sum_{S_{j}\in A_{S_{i}}}P\left(S_{j}\right)\right), \end{array}$$

where *H* is the Shannon entropy of the system and, with consideration of the log function, the second term has a negative value between −H and 0; see [12] where conditional Shannon entropy is similarly expressed by involving a mutual information term. For *U* and *R* the constant functions which everywhere take the value 1, (4) simplifies to *H*.

Because Shannon’s Information Theory is so well developed and useful it is interesting to consider whether expected float entropy can be recast to be fully compatible with Information Theory. The answer is yes although results of such a theory have yet to be obtained. To show this suppose we have a system with probability distribution $P:\Omega_{S,V}\to \left[0,1\right]$. For $R\in \Psi_{S}$ and $U\in \Psi_{V}$ we will define a partition $\hat{\Omega }\left(R,U\right)\subseteq 2^{\Omega_{S,V}}$ of $\Omega_{S,V}$. Such a partition $\hat{\Omega }\left(R,U\right)$ can be treated as a set of states of a random variable with

$$\begin{array}{c} \sum_{A\in \hat{\Omega }\left(R,U\right)}P\left(A\right)=\sum_{A\in \hat{\Omega }\left(R,U\right)}\sum_{S_{i}\in A}P\left(S_{i}\right)=\sum_{S_{i}\in \Omega_{S,V}}P\left(S_{i}\right)=1 \end{array}$$

and joint probability distribution

$$\begin{array}{c} P\left(S_{i},A\right)=P\left(\left\{S_{i}\right\}\cap A\right)=\left\{\begin{array}{cc} P(S_{i}) & \mathrm{if}S_{i}\in A,\\ 0 & \mathrm{otherwise}. \end{array}\right. \end{array}$$

Note that this gives the correct marginal distributions,

$$\begin{array}{c} \sum_{A\in \hat{\Omega }\left(R,U\right)}P\left(S_{i},A\right)=P\left(S_{i}\right)\;\mathrm{and}\;\sum_{S_{i}\in \Omega_{S,V}}P\left(S_{i},A\right)=\sum_{S_{i}\in A}P\left(S_{i}\right)=P\left(A\right), \end{array}$$

and allows us to apply the standard definitions from Information Theory such as joint entropy, mutual information and conditional entropy; see [12] for the Information Theory definitions mentioned. In particular, conditional entropy can be taken as a new definition of efe provided that $\hat{\Omega }\left(R,U\right)$ (the set of states of the random variable used in the condition) depends on *R* and *U* in a suitable way to give a low conditional entropy value when *R* and *U* give a low efe value. We introduce Algorithm 1 below to do this. To this end, suppose *R* and *U* minimise efe, as defined in Definition 5, and $S_{i}\in \Omega_{S,V}$ has a relatively large probability. Algorithm 1 below defines $\hat{\Omega }\left(R,U\right)$ such that in this case the unique set $A\in \hat{\Omega }\left(R,U\right)$ with $S_{i}\in A$ contains few other elements besides $S_{i}$ so that $\hat{\Omega }\left(R,U\right)$, as a set of states of a random variable, is similar to the set of states $\Omega_{S,V}$ of the random variable defined by the system. This will result in a low conditional entropy value when *R* and *U* minimise efe. Note that from the outset it is possible to use $\left\{S_{i}\in \Omega_{S,V}:P\left(S_{i}\right)>0\right\}$ in place of $\Omega_{S,V}$ without affecting conditional entropy.

**Algorithm 1:** Partition algorithm for when using conditional entropy similarly to efe.Step 1: Let $S_{i}\in \Omega_{S,V}$ be such that: (a)$S_{i}$ has yet to be allocated to one of the subsets $A\subseteq \Omega_{S,V}$ partitioning $\Omega_{S,V}$;(b)of all the elements of $\Omega_{S,V}$ satisfying (a), $S_{i}$ has the greatest probability;(c)for $A_{S_{i}}:=\left\{S_{j}\in \Omega_{S,V}:d\left(R,R\left\{U,S_{j}\right\}\right)\leq d\left(R,R\left\{U,S_{i}\right\}\right)\right\}$, if there is more than one element of $\Omega_{S,V}$ satisfying (a) and (b) then $S_{i}$ is such that $A_{S_{i}}$ contains the fewest elements. If $S_{i}$ is still not unique then choose any such $S_{i}$ that satisfies (a), (b) and (c). It will turn out that $\hat{\Omega }\left(R,U\right)$ is well defined because if $\#A_{S_{i}}=\#A_{S_{j}}$ then $A_{S_{i}}=A_{S_{j}}$. Step 2: Define the next subset of $\Omega_{S,V}$ contributing to the partition of $\Omega_{S,V}$ to be $$\begin{array}{c} \{S_{j}\in A_{S_{i}}:S_{j}\mathrm{has}\;\mathrm{yet}\;\mathrm{to}\;\mathrm{be}\;\mathrm{allocated}\;\mathrm{to}\;\mathrm{one}\;\mathrm{of}\;\mathrm{the}\;\mathrm{subsets}\;A\;\mathrm{partitioning}\;\Omega_{S,V}\}. \end{array}$$ Step 3: If all the elements of $\Omega_{S,V}$ have been allocated then define the partition $\hat{\Omega }\left(R,U\right)$ of $\Omega_{S,V}$ as the set of the subsets defined during each occurrence of Step 2. Otherwise, if there are still elements of $\Omega_{S,V}$ to be allocated then return to Step 1. 

Before moving on to Section 2 it is worth noting that the examples in reference [1] are intended to have relevance to the visual cortex and our experience of monocular vision. In the present article, in order to investigate the extent to which expected float entropy minimisation is an effective association learning process we will use typical data for digital photographs of natural scenes as training and testing data, although other data sources could have as easily been used such as auditory data. Expected float entropy minimisation will be used to determine relationships between pixel locations (recovering the spatial geometry of the photographs) and also relationships between the pixel states (recovering the similarity between similar shades of grey in the photographs). Subsequently, to test the effectiveness of expected float entropy minimisation as an association learning process, the learned relationships will be used to perform minimum float entropy completion. The test involves completing missing pixel states for a test set of photographs that each have 44% of their pixel state data involved removed. The performance of recovering missing pixel states by choosing those states that give the minimum float entropy value (relative to the relationships learned) is then compared with a state of the art black box using the neural fitting tool in MATLAB which is applied to the same data completion task.

For a discussion on how the theory under investigation solves the binding problem, see references [1,2]. In short, consciousness may largely be the state of the system in the context of the relationship parameters that minimise a version of expected float entropy that involves more than the two weighted relations used in Equation (1); see the discussion in Section 4 about multi-relational float entropy, the definition of which first appeared in reference [1].

The rest of the article is organized as follows. Section 2 looks at obtaining typical data from digital photographs, references the methods used for solving Equation (1), and describes efe-histograms. Section 3 tests how well expected float entropy minimisation performs as an association learning process. Section 4 discusses multi-relational float entropy, the inherent probability distribution of a system, experimental testing on the brain, and observable phenomenon and unobservable effects. The conclusion is given in Section 5. The appendix provides a list of notation.

## 2. Methods and Materials

In this section we look at obtaining typical data from digital photographs, references to algorithm for finding solutions to Equation (1), and using efe-histograms to assess guesses when guessing solutions to Equation (1).

### 2.1. Typical Data from Digital Photographs

When obtaining a typical data element from a digital photograph, in the present article, only a small part of the photograph is used. This is because the computational methods used in the present article are suitable for small systems $\left(\#\Omega_{S,V}\leq 10^{6}\right)$ although, at the expense of ease of implementation, other more efficient computational methods are possible for investigating larger systems.

**Figure 1:**
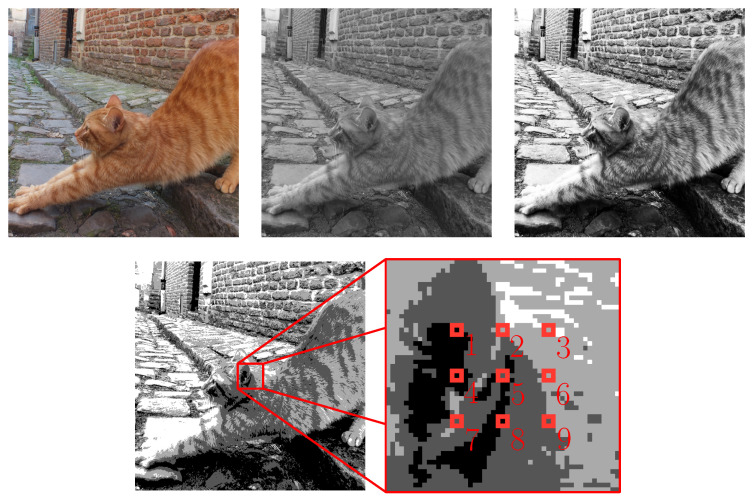
Digital photograph sampling using nine nodes and a four shade gray scale.

Figure 1 shows the sampling of a digital photograph such that the typical data element obtained is for a system comprised of nine nodes with a four state node repertoire $(\#\Omega_{S,V}=4^{9}=$ 262,144). Also, in the case of Figure 1, we are using pixel brightness to determine node state. From top-left to bottom-right, the first image is the original. This image is desaturated (the colours are turned into shades of gray) and then the contrast is enhanced. The contrast enhancement is not required, but it was thought that it might reduce the number of typical data element needed in order to obtain meaningful results. The image is then posterised (in this case the number of shades is reduced to four giving a four state node repertoire). Finally, nine pixels are sampled giving the state of each of the nine nodes of the typical data element; see Table 2.

To obtain the typical data for the system, this way of obtaining typical data elements is used for several hundred digital photographs of natural scenes of the world around us. One typical data element is obtained from each photograph. Importantly, what ever the geometric layout of the pixel sampling locations (in Figure 1 the layout is part of a grid that has adjacent locations every ten pixels), the same layout must be used for all of the digital photographs. Similarly, the same criteria must be used for determining the node states. Once the typical data for the system has been obtained, optimisation is used to find solutions to Equation (1) by using efe(R,U,T) as an objective function. This reveals which relationships *R* and *U* the system defines, respectively, between the nodes (recovering the grid geometry, which would extend to the whole 2D geometry of the images if all the pixels were used) and between the shades of gray recovering the gray scale. Note that the relationships solving Equation (1) depend exclusively on the bias of the system to certain states, and on the nodes involved, and are otherwise not given a priori. This is important for a theory for how a system like the brain is itself able to define the relationship content of consciousness; see Section 4.2. For solving Equation (1), a simple optimisation (binary search) algorithm is given in [1] and is available as part of the software URFinder 3.7 available from the author; see Supplementary Materials. Gradient decent may also be applicable, advanced optimisation methods are described in [13,14,15], and computationally faster surrogates for efe(R,U,T) can be obtained using machine learning.

### 2.2. Using Efe-Histograms Obtained from Monte-Carlo Methods

Here we choose $U\in \Psi_{V}$ and $R\in \Psi_{S}$ uniformly at random. With reference to Table 1, this is done by choosing each off-diagonal upper-triangular entry of *U* and *R* uniformly at random from the interval [0,1] (the off-diagonal lower-triangular entries are then those making *U* and *R* symmetric). The efe value is then calculated and stored, and the whole process is repeated producing a list of many thousands of efe observations from which an efe-histogram can be obtained. With this setup, if we wish to treat efe as a random variable then standard methods can be used for approximating the probability distribution from the efe values (although this can be difficult for distributions with very thin tails). In any case, provided enough observations are made, the efe-histogram can be used to help assess guesses when guessing approximate solutions to Equation (1). It is also suggest in [1] that node selections that result in efe-histograms with long left tails are most important; see Section 4.1, Section 5 and also details on base branching structure in [1].

## 3. Results

**Figure 2:**
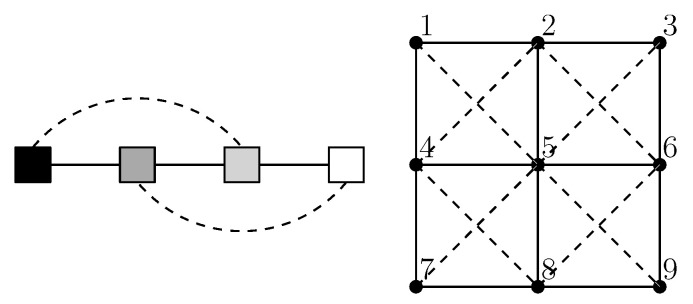
Graph illustration of the weighted relations in Table 3, showing strongest relationships (*solid lines*) and intermediate relationships (*dash lines*).

In this test of how well expected float entropy minimisation performs as an association learning process, 600 digital photographs of the world around us are used such that 400 are for training and 200 are for testing. The typical data is obtained using the method shown in Figure 1, where the photographs have a four shade gray scale. Hence, #T=400 and the system is comprised of nine nodes with a four state node repertoire giving $\#\Omega_{S,V}=4^{9}=$ 262,144. Similarly the data for the test set *W* is also obtained using the method shown in Figure 1 so that #W=200. For *T* a solution to Equation (1) was found for a subset of five of the nine nodes by using the binary search algorithm given in [1]. Using symmetry, the solution was then extended to all nine nodes as an approximate solution. The approximate solution is given in Table 3. Figure 2 provides a graph illustration of the weighted relations. For *U*, values above 0.2 are indicated with a solid line, whilst values from 0.02 to 0.2 are indicated with a dash line. For *R*, values above 0.9 are indicated with a solid line, whilst values from 0.75 to 0.9 are indicated with a dash line.

**Figure 3:**
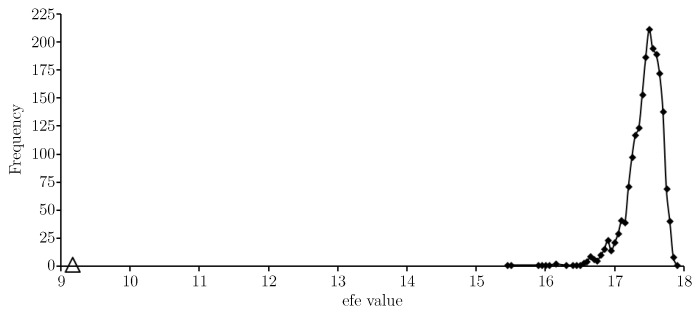
An efe-histogram for the training data using 2000 efe observations and a bin interval of 0.05. The efe value of the approximate solution is shown (*triangular marker*).

Figure 3 provides an efe-histogram for *T*. The efe value for the approximate solution is indicated with a triangular marker and shows the approximate solution gives a very low expected float entropy value relative to other choices.

Having completed the training stage we now look at testing. As is the case for *T* (see Definition 1), the test set *W* is a set of numbered observations. The elements of *W* are handled using a function ω (analogous to τ in Definition 1) where $S_{\omega (k)}$ is the data element representation of observation number *k* for k∈{1,…,200}. For each *k* we obtain an obfuscated version of $S_{\omega (k)}$ by choosing four of the nine nodes uniformly at random and deleting their state information. There are $4^{4}=256$ possibilities for how this obfuscated version of $S_{\omega (k)}$ can be completed and we denote these completions by $S_{\omega (k),i}$ for i∈{1,…,256}. Now using *U* and *R* obtained in the training stage (see Table 3), we obtain the set of numbered minimum float entropy completions of the obfuscated observations,

(5) $$\begin{array}{c} W_{\mathrm{mfe}}:=\left\{\left(k,i,S_{\omega (k),i}\right):1\leq k\leq 200,\mathrm{fe}\left(R,U,S_{\omega (k),i}\right)=\min_{1\leq j\leq 256}\mathrm{fe}\left(R,U,S_{\omega (k),j}\right)\right\}. \end{array}$$

**Figure 4:**
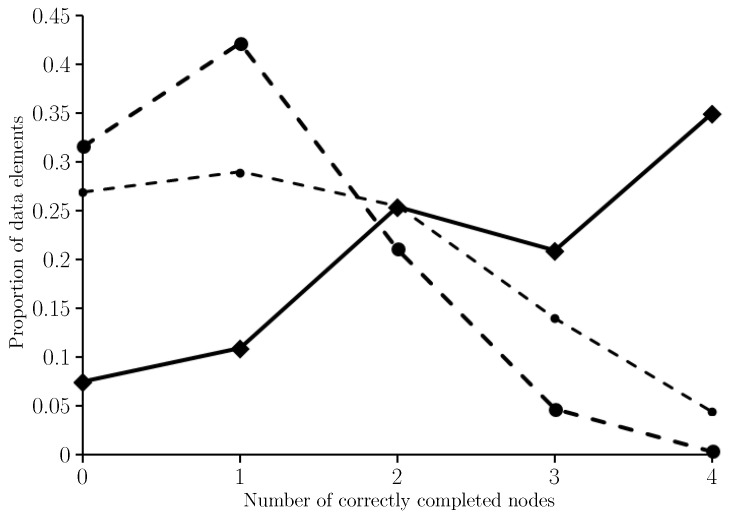
A histogram showing the proportion of the 200 test data elements that have *n* out of four nodes correctly completed, for n∈{0,1,2,3,4}, when using minimum float entropy completion (*solid line*) and when completing each node independently of the others by selecting for each node the most commonly observed state for that node in the training set (*light dash line*). For further comparison, the binomial distribution B(4,1/4) which gives the probability of correctly completing *n* out of four nodes when guessing node states uniformly at random for n∈{0,1,2,3,4} (*heavy dash line*).

The elements of $W_{\mathrm{mfe}}$ are numbered (that is they are made distinct by their k,i values) because the function ω is not injective. Moreover, $\#W_{\mathrm{mfe}}=202$ not 200 because for each of two particular values of *k* there were two values of *i* for which $\mathrm{fe}(R,U,S_{\omega (k),i})$ was the minimum float entropy value. The results of the test are given in Figure 4 where the proportion of the obfuscated observations that have *n* out of four nodes correctly completed are shown for n∈{0,1,2,3,4}.

In the case of the two obfuscated observations that have two completions in $W_{\mathrm{mfe}}$, the average number of nodes correctly completed was used and rounded down when not an integer. We see from Figure 4 that only 7.5% of minimum float entropy completions have no correctly completed nodes whilst 35% have all four nodes completed correctly. The average number of correctly completed nodes is 2.65. For comparison Figure 4 shows results for when completing each node independently of the others by selecting for each node the most commonly observed state for that node in the training set. In this case, the average number of correctly completed nodes is 1.4. Figure 4 also shows the binomial distribution B(4,1/4) which is the probability distribution for the number of correctly completed nodes if guessing the node states uniformly at random. In this case the expected number of correctly completed nodes is just 1.

Clearly minimum float entropy completion has performed far better than completing the nodes independently or just guessing node states uniformly at random. In fact the probability of this result happening by chance, under the assumption that minimum float entropy completion is no better than just guessing, is orders of magnitude smaller than $10^{-6}$. This was confirmed using the Normal approximation N(800(0.25),800(0.25)(1−0.25)) of the Binomial distribution B(800,0.25) for the total number of correctly guessed node states across all of the obfuscated elements of *W*. For $W_{\mathrm{mfe}}$ there are 530 correctly completed nodes out of 800 and statistics tables show that the probability of there being more than 260 correct completed node states when just guessing is less than $10^{-6}$.

Although we have proven that minimum float entropy completion performs far better than completing the nodes independently or just guessing, it is also right to compare it with a current state of the art learning process. For this we used the Neural Fitting Tool (nftool) in MATLAB which is for input-output fitting problems. To use nftool, an input set and a target set is needed for training, validation and testing. The union T∪W was taken as the target set and an obfuscated version of T∪W constitutes the input set, where, for each element of T∪W, we obtain an obfuscated version by choosing four of the nine nodes uniformly at random and deleting their state information. Four hundred input and target pairs were allocated to training and the remaining were used for validation and testing. Using a validation sample prevents over fitting by halting training when performance on the validation sample stops improving.

On the input side of the neural network, each of the nine nodes connect to four neurones that are dedicated to just that node, that is they do not have an input connection from any of the other nodes. Therefore there are 36 neurons in the first layer. This allows each of the node states to be represented in binary vector form with the following representations: black (1,0,0,0); dark gray (0,1,0,0); light gray (0,0,1,0); white (0,0,0,1); obfuscated (0,0,0,0).

Next, nftool has a two-layer feed-forward network with sigmoid hidden neurons and linear output neurons. Such neural networks can fit multi-dimensional mapping problems arbitrarily well given consistent data and enough neurons in its hidden layer. In our case the data is not consistent since the target set can have different completions for two different instances of the same input. It was found that having 40 neurons in the hidden layer gave the best results. The network is trained with the Levenberg-Marquardt backpropagation algorithm.

**Figure 5:**
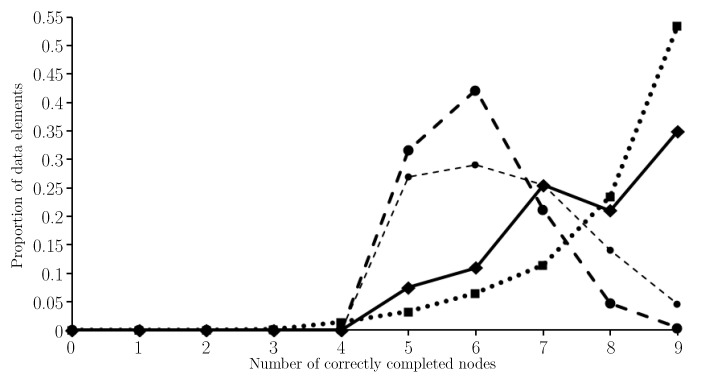
A histogram showing the proportion of the elements of the obfuscated version of T∪W that have *n* out of nine nodes correctly completed, when using nftool, for n∈{0,1,⋯,9} (*dotted line*). For comparison, the results shown in Figure 4 are included. The results when using minimum float entropy completion are shown (*solid line*), the results are shown for when completing each node independently of the others by selecting for each node the most commonly observed state for that node in the training set (*light dash line*), and the distribution for guessing node states uniformly at random is shown (*heavy dash line*).

As per the input side, there are four neurones for each of the nine nodes on the output side. However, a final max value layer is added so that a node output such as (−0.062,0.426,0.594,0.041) is converted to (0,0,1,0), representing light gray in this case. The results are given in Figure 5 where the proportion of the elements of the input set (which is the obfuscated version of T∪W) that have *n* out of nine nodes correctly completed are shown for n∈{0,1,⋯,9}. For comparison, the histogram shown in Figure 4 has also been included in Figure 5, all be it shifted to the right by five because minimum float entropy completion preserves the states of the specified nodes noting there are five nodes per data element with given state.

When using nftool the average number of correctly completed nodes was 8.13. When using minimum float entropy completion the average number of correctly completed (and preserved) nodes was 7.65. This average drops to 6.4 in the case of completing the nodes independently, and when guessing the obfuscated node states uniformly at random the expected number of correctly completed (and preserved) nodes is 6. Clearly nftool has outperformed minimum float entropy completion, although, for these two learning processes, the average number of correctly completed nodes per data element only differs by 0.48; note the difference is 1.73 when comparing the nftool performance with completing the nodes independently, and 2.13 when guessing.

In Section 4 we will discuss expected multi-relational float entropy minimisation which involves more than the two weighted relations used in Equation (1) and was first introduced in [1]. Although beyond the scope of the present article to demonstrate, additional relationship parameters should improve performance. In the case of the brain, multi-relational float entropy may reveal how the brain defines relationships between geometric structures in the field of view and, at a high level of meaning, between objects for example, noting that according to the theory, consciousness may largely be the states of the system in the context of the weighted relations that minimise expected multi-relational float entropy.

**Figure 6:**
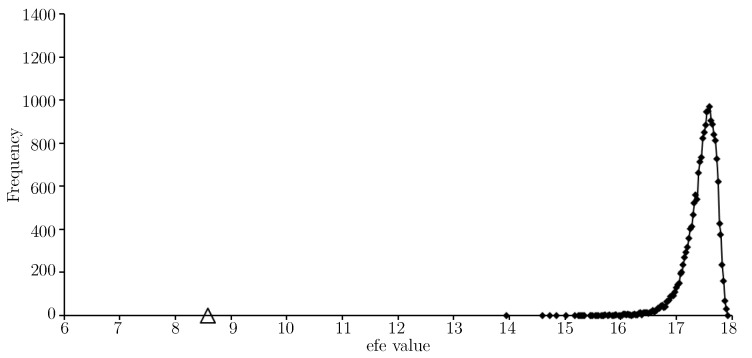
An efe-histogram for the test data as sampled using 20,000 efe observations and a bin interval of 0.025. The efe value of the approximate solution is shown (*triangular marker*).

Before moving on to Section 4 we now consider some efe-histograms (see Section 2.2) for the results presented in the present article. Figure 6 provides an efe-histogram for the test data *W* as sampled.

**Figure 7:**
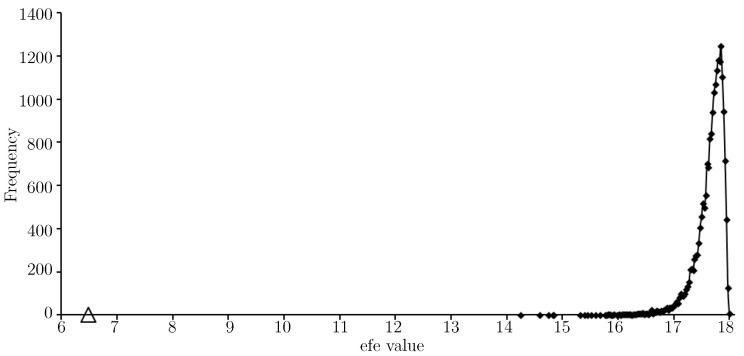
An efe-histogram for the minimum float entropy completed test data using 20,000 efe observations and a bin interval of 0.025. The efe value of the approximate solution is shown (*triangular marker*).

The efe value for the approximate solution (in Table 3) to Equation (1) is indicated with a triangular marker and shows the approximate solution gives a very low expected float entropy value (around 8.6) relative to other choices. In fact the value is slightly lower than that shown for the training set *T*, see Figure 3. Figure 7 provides an efe-histogram for the minimum float entropy completed test data.

**Figure 8:**
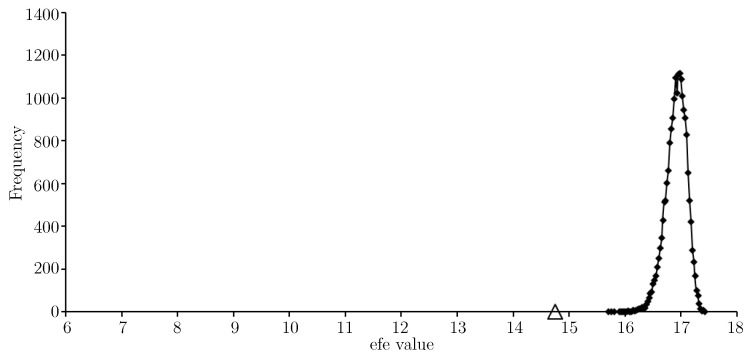
An efe-histogram for the uniform randomly completed test data using 20,000 efe observations and a bin interval of 0.025. The efe value of the approximate solution is shown (*triangular marker*).

Not surprisingly, the efe value given by the approximate solution in Table 3 is lower (now around 6.5) on the minimum float entropy completed test data than on *W*. Indeed, any discrepancy between the minimum float entropy completed test data and *W* will result in the efe value for the approximate solution being higher on *W*. For Figure 8 a set of completed test data was generated by choosing missing node values uniformly at random.

Completing the obfuscated test data in this way is a transformation of *W* that makes it more random. As per the discussion comparing expected float entropy and Shannon entropy in Section 1.1, the efe value of the approximate solution is much higher on the uniform randomly completed test data than it is on *W*. Finally we consider the nftool completed test data. In this case the efe value for the approximate solution (in Table 3) is very similar to what it is on *W*; around 8.5. For the same reason as for *W*, it is not surprising that this is higher than the result for the minimum float entropy completed test data.

## 4. Discussion

Section 3 shows expected float entropy minimisation is an effective association learning process and that minimum float entropy completion performed well at completing obfuscated observations. However, the results would likely be improved under expected multi-relational float entropy minimisation due to the additional relationship parameters involved. The following definition of multi-relational float entropy is slightly more general than the version given in [1].

**Definition** **6** (Multi-relational float entropy)**.** *Let S be as in Definition 1, let $U\in \Psi_{V}$, and let $R\in \Psi_{S}$. Furthermore, let $U_{1},U_{2},\ldots$ and $R_{1},R_{2},\ldots$ be weighted relations analogous to U and R but involving structures such as tuples of the initial nodes, subsets of the initial nodes and, ultimately, even objects, locations and places. The* multi-relational float entropy *of a data element $S_{i}\in \Omega_{S,V}$, relative to $U,U_{1},U_{2},\ldots$ and $R,R_{1},R_{2},\ldots$, is defined as*

$$\begin{array}{c} \mathrm{fe}(R,U,R_{1},U_{1},R_{2},U_{2},\ldots ,S_{i})\\ \;:=\log_{2}\left(\#\left\{S_{j}\in \Omega_{S,V}:C_{0}\left(R,U,R_{1},U_{1},R_{2},U_{2},\ldots ,S_{i},S_{j}\right)\wedge C_{1}\left(R,U,R_{1},U_{1},R_{2},U_{2},\ldots ,S_{i},S_{j}\right)\wedge \cdots \right\}\right), \end{array}$$

*where the first condition $C_{0}\left(R,U,R_{1},U_{1},R_{2},U_{2},\ldots ,S_{i},S_{j}\right)$ is $d\left(R,R\left\{U,S_{j}\right\}\right)\leq d\left(R,R\left\{U,S_{i}\right\}\right)$, as in Definition 5.*

In Definition 6, all of the conditions $C_{0},C_{1},\cdots$ need to be satisfied for a data element $S_{j}$ to contribute toward the multi-relational float entropy of a data element $S_{i}$. The additional conditions should be those that increase the length of the left tail of the efe-histogram.

**Figure 9:**
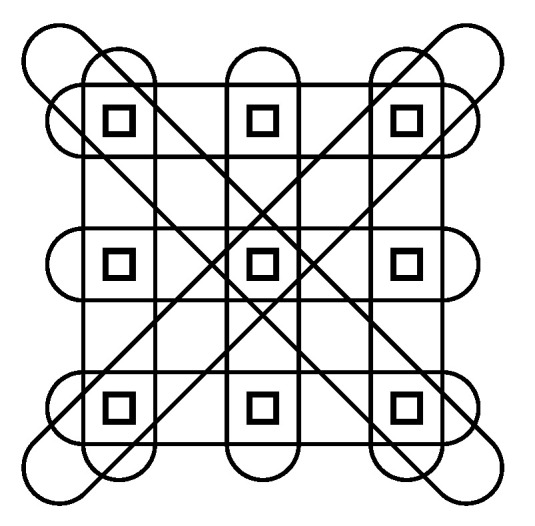
Eight tuples each containing three of the initial nodes. The geometric layout of the initial node is that of the sampling locations in Figure 1.

Let us consider an example that would likely improve the results obtained in Section 3 by adding an additional condition $C_{1}$ analogous to $C_{0}$ but involving tuples of the initial nodes. Figure 9 identifies eight tuples with each containing three of the initial nodes. Let $S^{\prime }$ denote the set of new nodes.

The repertoire of the new nodes is large $4^{3}=64$ but a much reduced repertoire $V^{\prime }$ can be used instead of $V^{3}$ to ease computation. For example a three state repertoire $V^{\prime }=\left\{v_{1}^{\prime },v_{2}^{\prime },v_{3}^{\prime }\right\}$ can be used such that a tuple in state $\left(v_{i},v_{j},v_{k}\right)$ is taken to be in state $v_{1}^{\prime }$ if i=j=k, state $v_{2}^{\prime }$ if $v_{i}$, $v_{j}$ and $v_{k}$ are not all the same but are strongly related according to *U* (e.g., they involve two adjacent shades in Figure 2), and state $v_{3}^{\prime }$ otherwise. The condition $C_{1}$ is $d\left(R_{1},R_{1}\left\{U_{1},S_{j}\right\}\right)\leq d\left(R_{1},R_{1}\left\{U_{1},S_{i}\right\}\right)$, where $R_{1}\in \Psi_{S^{\prime }}$ and $U_{1}\in \Psi_{V^{\prime }}$. Note that $C_{1}$ still depends on the elements of $\Omega_{S,V}$ because the states of the new nodes are a function of the states of the initial nodes. Minimising $\mathrm{efe}(R,U,R_{1},U_{1},T)$ will result in $R_{1}$ giving relatively strong relationships between parallel structures within *S* under the geometry determined by *R*. This is just one example of adding an extra condition but many conditions can be added and the possibilities are quite abstract. In this setup, states of new nodes need only be functions of the states of the initial nodes and don’t have to be tuples. Node states can be objects determined by the states of nodes in a particular location etc. With such wide possibilities it is right to ask how the system defines the conditions to be included. As mentioned, the additional conditions should be those that increase the length of the left tail of the efe-histogram. For a structure such as the brain, clues from the structure of the system itself should be sought. In particular the new nodes required may turn out to be within the system itself, in which case they can be included as initial nodes in *S* and conditions such as $C_{0}$ can be applied to different subsets of *S*. Multi-relational float entropy greatly extends the scope of the theory and more research is needed in this area.

It may be useful to mention how the computation time (assuming serial processing) for calculating a single efe value depends on the size of the system involved. With reference to Definition 5, the time taken to compute an efe value equals the number of typical data elements sampled, #T, multiplied by the time it takes to compute one float entropy value, which largely scales with the size of $\Omega_{S,V}$. Therefore, a first approximation of the computation time is given by $C\left(\#T\right)m^{n}$, where *n* is the number of nodes, *m* is the size of the node repertoire and *C* is a constant. It follows that the time increases linearly in #T, with polynomial time in *m* and with exponential time in *n*. If measuring time in seconds, *C* will typically be of order $10^{-6}$ or $10^{-7}$ depending on the contemporary processor used but will be larger for expected multi-relational float entropy where several conditions are involved. Note that calculating efe is an “embarrassingly parallel” task and lends itself to high performance computing methods.

### 4.1. The Inherent Probability Distribution a System Defines

According to the theory, the relationships a system defines are those that minimise expected (multi-relational) float entropy and this depends on the inherent probability distribution a system has. The theory, initially presented in [1], suggests that, to contribute to consciousness, a system will at least need an inherent probability distribution on its set of states that gives an efe-histogram with a long left tail because when the tail is very long the solutions to Equation (1) are isolated from other weighted relations and are therefore strongly determined by the system. Further, systems with an inherent probability distribution that is close to being uniform give a very short left tail and it is not expected that such uniformly random systems would give rise to consciousness. If a brain region processes information in a very compressed form then its inherent probability distribution will be more uniform than other regions and, according to the theory, regions contributing to consciousness should be found among those that are pre and post high compression. Further, as mentioned in the introduction, if the brain or some local region of the brain contributing to consciousness changes how it is functioning then this will change its inherent probability distribution over its states which will then change the relationships that minimise expected float entropy and consequently, according to the theory, the relational content of the experience. Such changes in functionality will occur due to the brain’s plasticity but also when activity patterns change as we transition from being awake to being in a deep sleep to being in REM sleep where activity patterns return to being similar to when awake. An important question to ask is when does a system actually define an inherent probability distribution, where the emphasis here is on the probability distribution being inherent as opposed to being applied by an external observer. For example, an external observer might associate audio compact discs (CDs) if they are in the same stack but this is a circumstantial association only and isn’t internal to any system. Similarly a television is rather like an electronic version of a flicker book of images and a flicker book is rather like a stack of images. On the other hand, the brain is a highly interconnected dynamical network which will snap to a state in response to a stimulus due to bias and learning. Understanding when an inherent probability distribution can be assumed for a system or subsystem is important for the presented theory and this issue might indicate a connection to other theories of consciousness such as Integrated Information Theory (IIT, see [16]) if a high Φ value were to correspond with the probability distribution being inherent. However, it is not necessary to answer this question in order to apply the theory, extract relationships and more generally accept expected (multi-relational) float entropy minimisation as a theory for the content of consciousness up to relationship isomorphism when consciousness is present. Also areas of the theory can be further explored and developed such as multi-relational float entropy without answering the question of when the probability distribution involved is inherent.

### 4.2. Experimental Testing on the Brain

In accordance with the scientific method, float entropy minimisation as a theory for uncovering the relationships a system defines needs to be tested on the brain noting it is propose that a brain state in the context of these relationships has meaning in the form of the relational content of the associated experience. The main obstacle to undertaking such testing is the lack of access to brain state data. In the future synthetic brains such as those sought by the Human brain project, if possible, would be able to provide the data needed but in the meantime less ideal tests are possible using FMRI data for the brain. One such possibility is to apply the theory to voxel data for layers within the visual cortex and possibly the Dorsal and Ventral streams. For example, the geometry of V1 cannot account for the perceived geometry of monocular vision because the retino-cortical mapping is approximately logarithmic and is far from being an isometry (see [17,18]) and signals from the right side of each retina are mapped to the right side of the brain, whereas signals from the left side of each retina are mapped to the left side of the brain. Despite the signals from the retina being split across two different brain regions in this way, the perceived geometry is a seamless isometric version of the image on the retina further enhanced by the brain’s abilities such as filling in. Applying expected float entropy minimisation to samples of voxel data for various parts of the visual system may recover the relationships that give the perceived geometry of the field of view. Note, the geometry could be very different from that of the actual physical locations of the voxel positions within the brain. However, difficulties may arise from the limited resolution of voxel data and also because the voxel states are not the neuron states but are instead a measure of blood flow. Therefore both the nodes and node repertoire involved are different to what we would be using if actual brain state data was available. However, the theory would identify relationships form voxel data and a thorough approach to the research could well produce valuable insights.

### 4.3. Observable Phenomenon and Unobservable Effects of Relationship Isomorphism

There are several different types of ambiguities that arise from the presented theory. Some of these ambiguities arguably result in observable phenomenon. In Section 3 a solution to Equation (1) was obtained and then minimum float entropy completion was used to complete missing node states for an obfuscated version of the observations. Two out of 200 of the obfuscated observations were able to be completed in two different ways under minimum float entropy completion. It is likely that the ambiguity in this case would disappear when using multi-relational float entropy but, more generally, ambiguities in the state a system should adopt under a given stimulation do occur in the form of bistable and multistable optical illusions. Another type of potentially observable ambiguity is due to the fact that expected float entropy minimisation only defines relationship parameters up to a certain resolution. Our experience of peripheral vision may be connected to this. We will now consider another type of ambiguity that we are unable to observe. According to the presented theory, expected (multi-relational) float entropy minimisation reveals relationships defined by the brain, and a brain state in the context of all these relationships acquires meaning in the form of the relational content of the associated experience. If the theory is correct then it explains how the brain defines the content of consciousness up to relationship isomorphism. It is important to note that this leads to an ambiguity because there can be several versions of consciousness that are relationally isomorphic to each other. An isomorphism is a form of symmetry. To formally clarify what is meant by relationship isomorphism, let $\left(X,R_{X},\ldots \right)$ and $\left(Y,R_{Y},\ldots \right)$ be two structures where *X* and *Y* are sets, $R_{X}$ and $R_{Y}$ are weighted relations on *X* and *Y* respectively and the dots represent any other attributes that the structures may have. If a bijective mapping α:X→Y is such that $R_{Y}\left(\alpha \left(a\right),\alpha \left(b\right)\right)=R_{X}\left(a,b\right)$ for all a,b∈X then α is a relationship isomorphism. In the case where the structures are not distinct, α is also a relationship automorphism. One potential example of relevance is the Inverted Spectrum hypothesis (which goes back to John Locke) which involves two people. Alice and Bob share the same colour vocabulary but one sees the inverse of the colours the other sees. Assuming that colour inversion is a relationship isomorphism, Alice and Bob will always agree on colours that are similar to each other and colours that are different even though they experience differently the colours they refer to. An alternative version of the Inverted Spectrum hypothesis (and a solution to the ambiguity of there being several relationally isomorphic versions of consciousness) is the hypothesis that Alice’s brain gives rise to several different versions of her consciousness with some versions seeing the inverse of the colours the others see. Each version of Alice will be unaware of the others and unable to access them because to do so would also affect colours when recollecting memories so that present experience always remains consistent with past experience. Another example of this involves the mirror image of the world around us. Mapping our experience of the geometry of the world around us to its mirror image is an isometry and therefore a relationship isomorphism. According to the hypothesis, some versions of Alice’s consciousness will see the mirror image of the world as perceived by the other versions. This is a very mathematical approach to resolving the problem of the ambiguity left behind by relationship isomorphism. When such ambiguities arise in mathematics usually the mathematical structure involved is enlarged to include the different possibilities and the isomorphisms involved form a group under composition. Issues involving mirror image mapping and relations have in the past been thought about by Kant and also arise in what is known as the Ozma problem. If the hypothesis that the brain defines several relationally isomorphic versions of consciousness is correct then a theory for how the brain defines the content of consciousness up to relationship isomorphism may be the best science can do. If the hypothesis is wrong then further theory may be possible.

## 5. Conclusions

The theory further investigated in this article brings together the facts that consciousness is full of relationships and that various brain regions determine an inherent probability distribution over their set of states which is not uniform due to learning processes that weaken or strengthen synapses. Hebb’s principle and its modern refinements are potentially examples of such learning processes and say that neurons increase the strength of their connections to neurons they are significantly influencing; see [6,7,8]. According to our theory, the link between these facts is made by expected (multi-relational) float entropy minimisation which takes the probability distribution of the system as an input along with relationship parameters of which a restricted choice is determined by the minimisation requirement. It is proposed that a brain state in the context of these relationship parameters has meaning in the form of the relational content of the associated experience. The theory, initially presented in [1], suggests that, to contribute to consciousness, a system will at least need an inherent probability distribution on its set of states that gives an efe-histogram with a long left tail. The length of the left tail may turn out to be of great importance because when the tail is very long the solutions to Equation (1) are isolated from other weighted relations and are therefore strongly determined by the system. Further, systems with an inherent probability distribution that is close to being uniform give a very short left tail and it is not expected that such uniformly random systems would give rise to consciousness.

In the present article we have established that expected float entropy minimisation is an effective association learning process. In particular, having used expected float entropy minimisation for training, minimum float entropy completion was compared with the Neural Fitting Tool (nftool) in MATLAB and also with completing the nodes independently as well as with completing missing node states uniformly at random. The nftool did outperform minimum float entropy completion but, for these two learning processes, the average number of correctly completed nodes per data element only differed by 0.48 where as the difference is 1.73 when comparing the nftool performance with completing the nodes independently, and the difference is 2.13 when guessing. Expected multi-relational float entropy minimisation will almost certainly give further improvements in performance and provides wide scope for further development of the theory involving many more relationships.

We have also shown that expected (multi-relational) float entropy minimisation can be recast to be fully compatible with Shannon’s Information Theory (see Algorithm 1) allowing us to apply the definitions of joint entropy, mutual information and conditional entropy. This also makes available a well developed body of theory that can be exploited in future work. In particular, conditional entropy minimisation can be implemented such that it should reveal similar relationships as those revealed using expected (multi-relational) float entropy minimisation. However, unlike expected (multi-relational) float entropy and (3), Algorithm 1 may place too much emphasis on the most probable states of the system and refinements may be needed. In Section 4.1 we noted the theory suggests consciousness is less likely to be found in brain regions that process highly compressed information. We noted functional changes affecting the inherent probability distribution a system defines will also affect the relational content of experience according to the theory. The issue of when a probability distribution is inherent to a system was also discussed. In Section 4.2 we put forward suggestions for how the theory could be tested on the brain using FMRI voxel data. In Section 4.3 we considered the possibility of there being a connection between bistable optical illusions and the occasional occurrence of there being more than one state satisfying minimum float entropy completion. We also considered the potential of there being unobservable effects of relationship isomorphism if the brain defines several relationally isomorphic versions of consciousness.

The overall conclusion is that expected float entropy minimisation is an effective association learning process but more research is desirable in to expected multi-relational float entropy minimisation. The theory and test results presented in this article are clearly consistent with the proposition of there being a close connection between association learning processes and consciousness. Whilst rather different in content, readers may also find [19,20,21,22,23] to be of interest.

## Figures and Tables

**Table 1:** Example of weighted relation tables.

*U*	$v_{1}$	$v_{2}$	$v_{3}$	⋯	*R*	Node 1	Node 2	Node 3	⋯
$v_{1}$	1	$u_{1,2}$	$u_{1,3}$	⋯	node 1	1	$r_{1,2}$	$r_{1,3}$	⋯
$v_{2}$	$u_{2,1}$	1	$u_{2,3}$	⋯	node 2	$r_{2,1}$	1	$r_{2,3}$	⋯
$v_{3}$	$u_{3,1}$	$u_{3,2}$	1	⋯	node 3	$r_{3,1}$	$r_{3,2}$	1	⋯
⋮	⋮	⋮	⋮	⋱	⋮	⋮	⋮	⋮	⋱

**Table 2:** Node states of the typical data element obtained from the sampling in Figure 1.

	Node 1	Node 2	Node 3	Node 4	Node 5	Node 6	Node 7	Node 8	Node 9
$S_{\tau (1)}$	0.000	294.449	294.449	0.000	147.224	294.449	147.224	0.000	147.224

**Table 3:** Approximate solution for *U* and *R*.

*U*	0	147.224	294.449	441.673					
0	1	0.29688	0.04688	0.01563					
147.224	0.29688	1	0.42188	0.10938					
294.449	0.04688	0.42188	1	0.32813					
441.673	0.01563	0.10938	0.32813	1					
*R*	node 1	node 2	node 3	node 4	node 5	node 6	node 7	node 8	node 9
node 1	1	0.95313	0.73438	0.95313	0.79688	0.60938	0.73438	0.60938	0.60938
node 2	0.95313	1	0.95313	0.79688	0.95313	0.79688	0.60938	0.73438	0.60938
node 3	0.73438	0.95313	1	0.60938	0.79688	0.95313	0.60938	0.60938	0.73438
node 4	0.95313	0.79688	0.60938	1	0.95313	0.73438	0.95313	0.79688	0.60938
node 5	0.79688	0.95313	0.79688	0.95313	1	0.95313	0.79688	0.95313	0.79688
node 6	0.60938	0.79688	0.95313	0.73438	0.95313	1	0.60938	0.79688	0.95313
node 7	0.73438	0.60938	0.60938	0.95313	0.79688	0.60938	1	0.95313	0.73438
node 8	0.60938	0.73438	0.60938	0.79688	0.95313	0.79688	0.95313	1	0.95313
node 9	0.60938	0.60938	0.73438	0.60938	0.79688	0.95313	0.73438	0.95313	1

## Supplementary Materials

The software URFinder3.7 is available online at http://www.jwmason.net.

## Abbreviations

The following abbreviations are used in this manuscript:

FE	Float Entropy
EFE	Expected Float Entropy

## Appendix A.

**Table A1:** Notation (most of the formal definitions can be found in Section 1.1).

Symbol	Description
α	a relationship isomorphism α:X→Y.
a,b,c,…	elements of *S* but also used to denote elements of other sets.
*A*	an element of $2^{\Omega_{S,V}}$. For clarification, *A* is just a subset of $\Omega_{S,V}$.
B(n,p)	the Binomial distribution.
$C_{0},C_{1},C_{2},\ldots$	conditions, involving weighted relations, in the definition of
	multi-relational float entropy.
d	a metric on the set of all weighted relations on *S* or, in places, a metric on
	${\left[0,1\right]}^{n}$.
$d_{n}$	for n∈N∪{∞}, a metric (on the set of all weighted relations on *S*)
	obtained from the corresponding *p*-norm, for p=n, on a finite dimensional
	vector space.
efe(R,U,P)	the expected float entropy, relative to *U* and *R*, of the given system.
efe(R,U,T)	the mean approximation of efe(R,U,P).
$\mathrm{fe}(R,U,S_{i})$	the float entropy, relative to *U* and *R*, of the data element $S_{i}$.
$\mathrm{fe}(R,U,R_{1},U_{1},R_{2},U_{2},\ldots ,S_{i})$	the multi-relational float entropy, relative to $U,U_{1},U_{2},\ldots$ and $R,R_{1},R_{2},\ldots$,
	of the data element $S_{i}$.
$f_{i}$	the map $f_{i}:S\to V$ corresponding to the data element $S_{i}$.
*H*	the Shannon entropy of the system.
node 1,node 2,node 3,…	elements of *S*.
N(np,np(1−p))	the Normal approximation of the Binomial distribution B(n,p).
*P*	the probability distribution $P:\Omega_{S,V}\to \left[0,1\right]$ of the random variable defined
	by the bias of the given system. *P* extends to a probability measure on $2^{\Omega_{S,V}}$.
*R*	an element of $\Psi_{S}$.
$R\{U,S_{i}\}$	the element of $\Psi_{S}$ given by the canonical definition
	$R\left\{U,S_{i}\right\}\left(a,b\right):=U\left(f_{i}\left(a\right),f_{i}\left(b\right)\right)$ for all a,b∈S.
*S*	a nonempty finite set; in most places *S* denotes the set of nodes of a system.
$S_{i}$	a data element for *S*, i.e., a system state given by the aggregate of the node
	states.
*T*	the typical data for the given system, i.e., *T* is a finite set of numbered
	observations of the given system.
τ	the map $\tau :\left\{1,\ldots ,\#T\right\}\to \left\{i:S_{i}\in \Omega_{S,V}\right\}$ for which $S_{\tau (k)}$ is the data
	element representation of observation number *k* in *T*. τ need not be
	injective.
*U*	an element of $\Psi_{V}$.
$v_{1},v_{2},v_{3},\ldots$	elements of *V*.
*V*	the node repertoire, i.e., the set of node states for a given system.
*W*	a finite test set of numbered observations of the given system.
$W_{\mathrm{mfe}}$	the set of numbered minimum float entropy completions of the obfuscated
	elements of *W*.
ω	the map $\omega :\left\{1,\ldots ,\#W\right\}\to \left\{i:S_{i}\in \Omega_{S,V}\right\}$ for which $S_{\omega (k)}$ is the data
	element representation of observation number *k* in *W*. ω need not be
	injective.
$\Psi_{S}$	the set of all reflexive, symmetric weighted-relations on *S*.
$\Psi_{V}$	the set of all reflexive, symmetric weighted-relations on *V*.
$\Omega_{S,V}$	the set of all data elements $S_{i}$, given *S* and *V*.
$\hat{\Omega }\left(R,U\right)$	a partition of $\Omega_{S,V}$ determined by *R*, *U* and, implicitly, *P*.
$2^{\Omega_{S,V}}$	the power set of $\Omega_{S,V}$.
